# Supplementary material for: Casein Glycomacropeptide: An Alternative Protein Substitute in Tyrosinemia Type I
Source: Nutrients. 2021 Sep 16;13(9):3224. doi: 10.3390/nu13093224 (PMC8467066; doi:10.3390/nu13093224)
Supplement: Supplementary file 1 [file nutrients-13-03224-s001.zip › nutrients-1363600-supplementary.pdf]

Supplementary tables

Table S1. **Pre study gastrointestinal symptoms.**

Patient ID	Heart burn	Laxative use	Abdominal discomfort	Nausea	Vomiting	Constipation
1.	0	0	0	0	0	0
2.	0	0	0	0	0	0
3.	0	0	0	0	0	0
4.	1	0	2	2	0	3
5.	1	0	0	0	0	0
6.	1	0	2	0	0	0
7.	0	0	0	0	0	0
8	0	0	0	0	0	0
9.	0	0	3	2	0	0

Abbreviations: 0 = no symptoms, 1 mild, 2 moderate, 3 severe, 4 very sever

Table S2. **Week 1 gastrointestinal symptoms using study product.**

Patient ID	Burping	Regurgitation	Abdominal bloating	Abdominal discomfort	Nausea	Vomiting	Flatulence	Constipation	Diarrhoea
1.	0	0	0	0	0	0	0	0	0
2.	0	0	0	0	0	0	1	0	0
3.	0	0	0	0	0	0	0	0	0
4.	0	1	1	0	1	0	0	1	0
5.	1	1	0	0	0	0	1	0	0
6.	1	0	0	2	0	0	1	0	0
7.	0	0	0	0	0	0	0	0	0
8.	0	0	0	0	0	0	0	0	0
9.	0	0	1	2	1	0	0	0	0

Abbreviations: 0 = no symptoms, 1 mild, 2 moderate, 3 severe, 4 very sever

Table S3. **Week 4 gastrointestinal symptoms using study product.**

Patient ID	Burping	Regurgitation	Abdominal bloating	Abdominal discomfort	Nausea	Vomiting	Flatulence	Constipation	Diarrhoea
1	0	0	0	0	0	0	0	0	0
2	0	0	0	0	0	0	1	0	0
3	0	0	0	0	0	0	0	0	0
4	0	0	0	0	0	0	0	0	0
5.	1	0	0	0	0	0	1	0	0
6.	1	0	0	0	0	0	1	0	0
7.	0	0	0	0	0	0	0	0	0
8.	0	0	0	0	0	0	0	0	0
9.	0	1	0	0	0	0	0	0	0

Abbreviations: 0 = no symptoms, 1 mild, 2 moderate, 3 severe, 4 very sever

Table S4. **Palatability Likert scale.**

Patient ID	Appearance	Smell	Taste	Aftertaste	Texture	Packaging	Ease of use
1.	5	5	4	5	5	3	3
2	5	3	3	3	5	3	3
3	5	4	4	3	3	2	3
4.	4	4	5	4	4	4	4
5.	3	4	3	2	3	2	3
6.	2	3	4	5	2	3	3
7.	3	4	4	4	3	3	3
8.	3	4	4	4	3	3	3
9.	3	4	3	3	3	3	3

Abbreviations: 1 dislike it at lot, 2, dislike it, 3 neither like or dislike it, 4 like it, 5 like it a lot
